# Adaptation of the Content of a Behavioural Text Message Delivered Weight Management Intervention for a Socio‐Culturally and Geographically Diverse Population of Postpartum Women in the UK: The Supporting MumS (SMS) Intervention

**DOI:** 10.1111/hex.70368

**Published:** 2025-08-06

**Authors:** Eleni Spyreli, Lizzie Caperon, Emma Ansell, Sara Ahern, Sally Bridges, Elinor Coulman, Stephan U. Dombrowski, Frank Kee, Jayne V. Woodside, Dunla Gallagher, Michelle C. McKinley

**Affiliations:** ^1^ Centre for Public Health Queen's University Belfast Belfast Northern Ireland UK; ^2^ Bradford Institute for Health Research Bradford England UK; ^3^ Centre for Trials Research (CTR) Cardiff University School of Medicine Cardiff Wales UK; ^4^ Faculty of Kinesiology University of New Brunswick Fredericton New Brunswick Canada

**Keywords:** cultural adaptation, personal and public involvement, postpartum period, weight management intervention

## Abstract

**Background:**

The Supporting MumS (SMS) intervention, originally piloted in Northern Ireland, United Kingdom (UK), uses automated text messages aiming to support diet and physical activity behaviour change for weight management in the postpartum period. Before testing the effectiveness of the SMS intervention in a UK‐wide randomised controlled trial, it was important to ensure that the core component of the intervention was acceptable and culturally relevant for a diverse range of women across different regions of the UK.

**Objective:**

to undertake Personal and Public Involvement (PPI) to adapt the content of the previously developed library of text messages for a socio‐culturally and geographically diverse population of postpartum women.

**Setting and Participants:**

Recruitment focused on mothers who lived in London, Bradford and various locations in Scotland, who had had a child within the last 2 years and had struggled with their weight. Existing PPI networks and community groups helped identify PPI representatives.

**Design:**

The PPI activities employed an iterative process including three stages: (1) an online group session to review some of the text messages and provide immediate feedback; (2) online group sessions to review adaptations made to messages; and (3) working remotely on a one‐to‐one basis with PPI collaborators to review and provide comments and suggestions on the entire text message library (previously modified based on feedback from stages 1 and 2).

**Results:**

A total of 19 PPI representatives responded to the invitation and 18 contributed to the review of the SMS text messages: *n* = 12 from England [*n* = 4 from London (African‐Caribbean ethnicity); *n* = 8 from Bradford (Asian ethnicity]; *n* = 6 from Scotland (White ethnicity). During a period of 9 months (July 2021‐March 2022), they provided unprompted, positive feedback about the overall concept of a text message‐delivered intervention to support postpartum weight management. During review and discussion of the original text message content they suggested minor amendments on the length, language, humour and cultural relevance of the text messages. Overall, no messages needed major re‐writing.

**Conclusion:**

This PPI work provided useful suggestions for the cultural and regional adaptation of the content of a text message library that aims to support postpartum weight management. Minor modifications to the messages were suggested. The effectiveness of the Supporting MumS intervention will be tested in a UK‐wide trial.

**Patient or Public Contribution:**

Our PPI collaborators were identified through existing PPI networks and community groups. They contributed through online group sessions and on a one‐to‐one basis through email correspondence. They offered valuable insights into ways of enhancing the cultural and regional relevance of a library of text messages to support diet and physical activity behaviour change for weight loss and weight loss maintenance in the postpartum period.

## 1 Introduction

Globally, one in six pregnant women live with obesity, a number that has steadily increased over the past two decades [1]. In the United Kingdom (UK), approximately one in five women present with a Body Mass Index (BMI) ≥ 30 kg/m² at their first antenatal appointment [2]. Entering pregnancy with obesity heightens the risk of adverse health outcomes for both mother and child [3, 4, 5, 6] and increases the likelihood that the child will develop obesity later in life [7, 8], perpetuating an intergenerational cycle of obesity [9, 10].

Excessive gestational weight gain (i.e. gaining more weight than recommended during pregnancy) and postpartum weight retention (i.e. not losing all weight gained during pregnancy) are significant contributors to developing life‐long obesity [11, 12, 13, 14]. The postpartum period offers an opportunity to provide support for weight management however, qualitative research highlights the many challenges and pressures facing women at this time in their lives [15, 16, 17, 18]. This often means their own health and care needs are not the primary consideration as they prioritise care of their family but, nevertheless, women do express a desire for support to help them get back to feeling like themselves [19]. Many challenges inherent to motherhood, such as sleep deprivation, using food as short‐term emotional support, lack of time, financial worries and mental health concerns, can impede the ability to maintain a balanced diet and engage in physical activity [15, 16, 17, 18] and careful consideration must be given to the design of interventions for women at this stage of life [19].

The Supporting MumS (SMS) intervention was developed with consideration of the specific needs of women in the postpartum period in both the intervention and trial design [20] with the aim of testing the delivery of postpartum weight management support via text messages. The full rationale and evidence base that was used to inform the development of the intervention has been previously described [20]. The SMS text message library was developed in close consultation with a group of postpartum women in Northern Ireland using an iterative process of writing and feedback with a focus on getting the length, tone and content of the messages right for the target audience [20]. After a year developing the intervention content with Personal and Public Involvement (PPI), it was tested for feasibility and acceptability in a two‐arm, pilot randomised control trial (RCT) in Northern Ireland, UK. Participants (*n* = 100 women who had given birth in the previous 2 years, BMI ≥ 25 kg/m^2^) were randomised to receive either the intervention text messages focused on diet and physical activity with embedded behaviour change techniques known to be positively associated with weight management, or text messages about child health and development (active control). The 12‐month pilot RCT showed that the SMS intervention was acceptable and feasible to implement and supported progression to a UK‐wide trial to examine effectiveness and cost‐effectiveness [20].

The SMS full trial aims to recruit a geographically, ethnically and socio‐demographically diverse sample of women from across the UK [21]. The text message library was initially developed and tested with a Northern Irish population from a predominately White ethnic background. Health communication needs to be sensitive to variations in culture to enhance reach and effectiveness [22]. There are linguistic variations across different regions of the UK and nuances around language, such as humour and idioms, that are shaped by culture. Dietary choices, preferences and other health behaviours are influenced by many factors including taste, cost, availability and also, importantly, by cultural values, norms and beliefs. Interventions designed for one target population may, therefore, not be relevant or acceptable to others if they aren't adapted to consider other cultural heritages and practices. Within behaviour change interventions, this means ensuring the language used and specific content, such as types of food and physical activity, are relevant to multiple cultures.

Health inequalities are strongly related to income, ethnicity and geography. In relation to diet and physical activity, Asian and Black women living in the UK are less likely to be physically active (i.e. for ≥ 150 min/week) and to meet the ‘5 a day’ recommendation compared to white women [23, 24]. Moreover, individuals on lower household incomes have a lower intake of fruit, vegetables and oily fish and higher intakes of sugar‐sweetened drinks compared to those on higher income levels [25]. Health inequalities have also been attributed to geography, with marked differences in life expectancy and disability prevalence across the four countries of the UK (England, Northern Ireland, Scotland and Wales) [26].

Examining the effectiveness of the SMS intervention in a diverse sample of women is important from a health inequalities perspective to ensure interventions do not widen inequalities and for determining generalisability of the intervention for different population groups to inform implementation and scale‐up. In line with the ADAPT guidance, adapting the SMS intervention content for different sociocultural groups and regions across the UK before RCT roll‐out can ensure that the advice given as part of the intervention is relevant and sensitive to the needs of a wide range of women independent from ethnicity and income background [27]. Therefore, before embarking on the effectiveness trial, it was important to conduct further PPI to review the content of the SMS intervention text message library that had been developed for the pilot trial.

To undertake PPI work to review and adapt the content of the core component of the SMS intervention, a library of text messages to support diet and physical activity behaviour change for weight loss and weight loss maintenance, so that it is relevant and appropriate for a geographically, socioeconomically and culturally diverse population of women in the postpartum period.

## 2 Materials and Methods

### 2.1 Selection of PPI Representatives

The selection criteria for participation in this PPI work were

1. having had a child within the last 2 years;
2. struggling with own body weight; and
3. living in Southeast London with an African or Caribbean background; or living in Bradford with an Asian background; or living in various locations in Scotland (ethnicity not specified). The reason for targeting those areas was to capture a socioculturally diverse group of mothers from different parts of the UK, as the initial text message library had been developed with a Northern Irish population from a predominately White ethnic background. Given the similarity in ethnicity and migration trends in Scotland and Wales [28, 29], resources were prioritised to include one of these countries alongside the two areas in England.

Representatives were not screened or asked to provide any information on their anthropometric measurements (body weight/height) or their child's age for the purpose of this study.

PPI recruitment focused on attaining geographical, sociodemographic and ethnic diversity, specifically within areas that would be acting as recruitment sites for the subsequent effectiveness trial in England (London and Bradford) and various locations in Scotland. PPI representatives were sought through several different avenues. In Bradford, mothers who were members of an established PPI network (Community Research Advisory Group) and had been previously involved in co‐designing research were approached. In Scotland, the invitation was shared with members of a PPI network of University of Stirling, who then circulated the invitation to relevant online forums of mothers which they had access to. In London, community organisations who worked closely with mothers were contacted and asked to circulate the invitation for participation to their service users.

A poster and information sheet were designed for recruitment (see Supporting Figures 1 and 2). The documents included information on what the PPI work required, as well as the contact details of the research team so that interested mothers could get in touch to express their willingness to take part or to ask questions and details on payment.

### 2.2 Text Message Library Content Adaptation

The PPI work conducted to develop the original library of text messages during the pilot phase guided the PPI approach for the cultural adaptation [20]. To fit this study within the busy lives of women who had recently had a baby we used flexible approaches that allowed us to gather detailed feedback while offering a range of options for collaboration (online calls, email and post), in a way that was convenient for the women involved. The adaptation work employed an iterative process with the aim of allowing PPI collaborators' feedback to inform the next steps of every activity.

There were three main stages to the PPI work starting with initial discussions in small groups then working with individuals later in the process.

### 2.3 Stage 1 (Group)

The first stage were group sessions, where the PPI representatives were introduced to the SMS study and the aim of the PPI exercise and were then asked to review a selection of text messages. The session was held in an online environment through safe teleconferencing platforms (MS Teams with PPI collaborators from London, Zoom with those from Bradford) and was directed using a discussion guide that was drafted in advance. During these sessions the facilitator presented a power point presentation with 32 messages using the share function of the meeting software and participants voiced their immediate feedback for each of the text messages. Each group consisted of 4–5 attendees and participants were encouraged to actively contribute their opinions on every message. Simultaneously, the facilitator ensured that all participants had the opportunity to share their feedback. The messages selected for the first stage were determined by the research team and covered topics that were considered particularly relevant when adapting content for different ethnic and cultural backgrounds, like social occasions, cooking methods, recipes and colloquialisms. Mothers reviewed the messages one by one and shared their thoughts on language, humour, clarity and cultural relevance. The session was recorded through the MS Teams recording function following receipt of verbal agreement from attendees. Obtaining the recordings ensured that the researchers could listen back to participants' feedback and take written notes which helped them review the text messages following the online sessions. The recordings were destroyed when complete notes were compiled for each session. Examples of text messages that were reviewed at this stage can be seen in Supporting Information S1: Figure 3.

### 2.4 Stage 2 (Group)

The text messages discussed at stage 1 were adapted by the research team based on the feedback of PPI collaborators. The same participants were then invited to a second online group session where they were asked to review the adapted messages. The facilitator sought their opinion on whether the updated messages were clearer, sounded more relevant and inclusive and, when that was not achieved, participants were invited to suggest alternative wording.

Additionally, at this session, PPI collaborators were asked to review a block of 3 weeks of text messages to get a feel for the flow and volume of messages as they would be received by all the participants of the SMS trial. As before, mothers discussed the messages and shared their thoughts on language, humour, clarity, length and cultural relevance. Similarly to stage 1, the audio recordings of the sessions were saved and used for compiling written notes of participants' feedback.

### 2.5 Stage 3 (Individual)

Based on the discussions from stage 1 and 2 and the nature and extent of the changes required, the PPI approach moved to working on an individual basis with representatives who agreed to review the entire library of text messages originally developed for the pilot study (some of these text messages had been previously reviewed and modified in stages 1 and 2) [20]. All PPI collaborators who took part in stage 2 were verbally invited to take part in stage 3. Those who agreed to participate were remotely based and were given the option to receive the lists of messages through post or email, or go through them verbally with a researcher. Again, the purpose was to provide PPI collaborators with a range of options, allowing them to choose the approach that best fitted with their everyday lives.

The work in this stage was completed in two steps. First, PPI collaborators were provided with the list of text messages to be delivered to all SMS participants in the first 12 weeks of the SMS intervention. Following feedback, PPI collaborators were sent the remaining text messages (from week 13–52). Before sending messages out, the messages were reviewed by the research team and modified in line with previous PPI feedback related to the similar content of other messages.

The messages were presented in a Word document (see Supporting Information S1: Figure 4) that included all text messages grouped by week number and listed in chronological order as they would be received in the intervention delivery. Next to each message, there was space provided for participants to indicate whether they liked/disliked the message and to note any additional changes in wording that they would recommend. All notes, including those from stage 1 and 2, were compiled with the feedback received from all PPI collaborators. The research team reviewed the notes and identified common areas for amendment that were then applied to the entire text message library. When adapting the wording of the text messages, careful consideration was given to ensuring that the changes retained the delivery of the embedded behaviour change techniques within the messages.

The rates of reimbursement were initially indicated in the participant information sheet (Sup. File 2) and were based on the time devoted to every task in accordance with the INVOLVE guidance for participation in PPI [30]. Specifically, PPI collaborators who took part in stage 1 and 2 were reimbursed with a £25 gift card per session, whereas those who reviewed the entire library of text messages at stage 3 received a £50 gift card per document. Payment was provided following the completion of each task.

## 3 Results

The three PPI stages are summarised in line with the reporting recommendations in Guidance for Reporting Involvement of Patients and the Public (GRIPP) [31]. The GRIPP2 checklist can be found in the Supporting Information S1: Table 1.

A total of 19 mothers responded to our invitation and 18 became involved:

- *n* = *12* from England:
  º *n* = 4 from London (African‐Caribbean), signposted to the PPI work by research team contacts within the community (maternity voices partnership)
  º *n* = 8 from Bradford (Asian), who were members of an established PPI network and directly contacted by the research team
- *n* = 6 from Scotland (White), who saw the invitation to participate in the online forums for mothers

Twelve representatives joined the online discussions in stage 1 and 2 (London (*n* = 4), Bradford (*n* = 8)). Twelve provided individual feedback on the entire text message library in stage 3 (London (*n* = 3), Bradford (*n* = 3), Scotland (*n* = 6)) and all of them opted to receive the lists of messages via email. The online group sessions lasted for approximately 1 h. The duration of all PPI activities was 9 months, from July 2021 to March 2022.

### 3.1 Overall Feedback on the SMS Intervention

Without prompting from the facilitator about the intervention concept, most participants expressed their interest in the idea of the SMS intervention and confirmed the need for such interventions. They commented on the friendly and empathetic tone of the text messages and were appreciative that the messages acknowledged the hectic schedule that comes with being a mum.

Early motherhood was described as an ‘isolating time’ highlighting the value of an intervention that considers a mother's need for support and positivity.

Suddenly you're at home with the baby and you no longer have that engagement with other adults. Yes, it would be quite important to get a message like that that would be uplifting and then make you think about you know, improving your mental health, as well as your physical.

(PPI in Scotland)

PPI collaborators agreed that the content of the messages adequately addressed common barriers to weight loss during the postpartum period and they felt the messages would provide them with encouragement and support not to give up on their weight loss goals. Participants liked the practical tips and the links to websites with healthy recipes, quick work‐out videos or location‐specific family‐friendly activities. Some of them underlined the value of being signposted to evidence‐based information sources within the text messages thus overcoming the issue of having to search for and filter the information themselves.

I searched and searched for a central thing, where you can find out all the classes that are going on and my God it has just come up as blank blank blank … so having something like that is great, because then it saves me from searching and I know a lot of moms don't have time.

(PPI in Bradford)

Finally, they felt that messages that included tips from mothers, which were based on previous PPI work conducted as part of the initial development of the library, added a personal tone that was received favourably.

### 3.2 Specific Suggestions for Text Messages

During Stage 1, 12 out of the 32 text messages discussed were positively received and were left unchanged. During all three stages, approximately 15% of the total messages (across all 3 stages) required no changes. No messages were considered to need major re‐writing (i.e. considered not appropriate or that it was very unclear and could not be understood unless modified). The minor amendments suggested during the PPI activities can be summarised in the following categories in descending order of frequency of suggestion based on PPI discussions:

1. shorten texts where possible;
2. avoid culturally and regionally specific colloquialisms that might not be widely understood;
3. avoid language that can be interpreted as judgemental;
4. avoid downplaying the effort required for weight loss;
5. avoid sensitive terms;
6. include more humour and emojis;
7. consider more culturally appropriate suggestions for food and exercise tips; and
8. if a message urges participants to act, clearly state its purpose.

These eight topics were all raised in PPI stages 1 and 2 and no new issues emerged during PPI stage 3. The suggestions are described in greater detail in the following paragraphs along with examples of reviewed messages, PPI feedback on how they should be amended and the final adapted message. Representative quotes with feedback on specific text messages, organised under each one of the eight topics, can be found in Table 1.

**Table 1:** Representative quotes from PPI collaborators with feedback on specific text messages, organised under eight topics.

1. Shorten texts where possible		
Original text message “Birthdays? Holidays? Weddings? Do any situations throw your healthy eating plans off track? You're not alone! Sometimes the best tactic is to accept it, and then plan ahead. If you know you will eat more on a family weekend away, make a plan (to get back to your healthy new lifestyle on the Monday).”	Feedback *Helpful but too much to read. Should be shortened and made ‘punchier’* *(PPI in Bradford)* *When you've just had a baby, the last thing you have time for is to constantly look at your mobile phone because there's so many things going on*. *(PPI in London)*	Adapted text message “Birthdays, weddings and other social situations may sometimes throw your healthy eating plans off track. Don't beat yourself up and enjoy them! Even if you happen to eat more than you intended, just get back to your healthy new habits afterwards.”
2. Avoid culturally specific colloquialisms that might not be widely understood		
Original text message “Tired and **time tied?** Dinner plans can sometimes **go out the window**! But who says you have to make a fancy meal? Pasta and pesto, oven chips and fish fingers (with some frozen or tinned peas/beans) make quick and handy dinners.”	Feedback *I don't understand the expression ‘time tied’, how about rephrasing it to ‘Tired and busy?’ I also think the expression ‘Go out of the window’ needs changing*. *(PPI in Bradford)*	Adapted text message “Tired and **no time**? Dinner plans can sometimes **fall apart**! But who says your meal has to be complicated or take ages to cook? Pasta and pesto, scrambled eggs and wholemeal pitta bread, oven chips and fish fingers (with frozen or tinned peas/beans) make quick and handy dinners.”
3. Avoid language that can be interpreted as judgemental		
Original text message “Are takeaways your to‐go option if you're short on time? Takeaways are handy once in a while, but **too many won't be good for your waistline**. In the time it takes to order a takeaway, you could make: vegetable/chicken stir‐fry, a baked potato with tuna, beans on toast or even a frozen veggie pizza, if those won't hit the spot.”	Feedback *Remove the ‘won't be good for your waistline’. At the end say something encouraging them to remain on track instead of reminding them that's not good for them. It's a bit judgy*. *(PPI in London)*	Adapted text message “Are takeaways your to‐go option if you're short on time? Takeaways are handy once in a while, but in the time it takes to order and get a takeaway, you could make: vegetable/chicken stir‐fry, baked potato with tuna, beans on toast or even a frozen veggie pizza. **If you crave a takeaway, why don't you try your own version? Here's a link to popular** ***fakeaways*** **:** [link]”
4. Avoid downplaying the effort required for weight loss		
Original text message “Have you slipped up this week? Don't worry, everyone does. **Just get back on track!** As the saying goes ‘if at first you don't succeed, **try, try again**’. Do you know what went wrong? How can you move forward? Check out this link for more tips on how to deal with slip‐ups: [link].”	Feedback *The ‘just get back’ makes it sounds too easy and if it was that easy, it wouldn't happen to begin with. Maybe take out the ‘just get back on track’ and after' do you know what went wrong’, put the ‘why don't you let us help you get back on track’…* *(PPI in London)* *I like the phrase ‘try and try again’*. *(PPI in London)*	Adapted text message “Have you slipped up this week? Don't worry, everyone does! As the saying goes ‘if at first you don't succeed, **try, try again’**. Do you know what went wrong? **Let us help you get back on track** ‐ check out this link for more tips on how to deal with slip‐ups: [link]”
5. Avoid sensitive terms		
Original text message “Cook yourself **thin**—home cooked meals can have up to 50% less calories than meals you have when eating out! 20 min can be all it takes: e.g. tuna pasta bake, with peas. Click here for recipes: [link].”	Feedback *The word ‘thin’ —I don't like it. I'm never going to be thin, it is a bit leading*. *(PPI in London)* *It can be a bit triggering in terms of weight loss. (PPI in London)*	Adapted text message “**Don't give up on cooking**! Home cooked meals can have up to 50% less calories than meals you have when eating out. 30 min can be all it takes. E.g. fish fingers, chicken and pasta salad, omelette and soups. Click here for recipes: [link]”
6. Include more humour and emojis		
Original text message “Already active? Great, stick with it. If not, don't fear, **we're not all born to be yoga bunnies or gym goddesses!** If the thought alone of being active makes you break into a sweat, stay tuned for easy and enjoyable ways to start moving.”	Feedback *I like the humour of it* *(PPI in London)* *Not sure about ‘yoga bunnies and gym bunnies’. The messages is understood but unsure how appropriate?* *(PPI in Bradford)*	Adapted text message “Already active? Great, stick with it. If not, don't fear, **we're not all born to be gym bunnies!** If the thought alone of being active makes you break into a sweat, stay tuned for more texts with easy and enjoyable ways to start moving.”
7. Consider more culturally appropriate suggestions for food and exercise tips		
Original text message “Whether you like it or loathe it, shopping with a baby can be challenging! **Why not try the ‘Click and Collect’ service** offered by most supermarkets? Save time, and **collect your groceries** when it suits you!”	Feedback *Click and collect might not be culturally appropriate for the Asian community*. *(PPI in Bradford)*	Adapted text messages “If you find that shopping with a baby is challenging, **why not try the ‘Shop Online’ service** offered by most supermarkets? Save time, plan ahead and **get your groceries collected or delivered** when it suits you. **Plus, online shopping can help you stay focused on what you really need!”**
8. If a message urges participants to act, clearly state its purpose		
Original text message “Made any changes to what you drink since the start of SMS? Maybe you're drinking more water or are having a smaller glass of vino? Text us… ”	Feedback *Is that to respond what they changed? Not clear what to text*. *(PPI in London)* *What is the purpose of this message? Not clear. Is it an assessment tool for the woman or is feedback for the project?* *(PPI in Bradford)*	Adapted text message “Made any changes to what you drink since the start of SMS? Maybe you're drinking more water, or cutting down on fizzy drinks or having a smaller glass of wine?” **Text us with any changes, we'd love to know!**

### 3.3 Shorten Texts Where Possible

PPI collaborators pointed out that, living in the era of social media, mothers are used to short bouts of information that they can quickly absorb then swiftly move to the next task. Therefore, long texts with repetitive content would be disregarded and remain unread. Messages targeting mothers should therefore be short, snappy and easy to read and understand within a few seconds.

### 3.4 Avoid Culturally and Regionally Specific Colloquialisms That Might Not be Widely Understood

PPI collaborators drew attention to regional variations of colloquial language and to some texts that used metaphors and idioms which might not be understood by all women across the UK (including nonnative English speakers). Examples of such phrases were ‘take the bull by the horns’ and ‘run out of steam’. PPI representatives pointed out that the use of such idioms in the text messages may compromise the clarity of the message and therefore, it was suggested they be replaced with a simpler, more literal wording.

### 3.5 Avoid Language That Can be Interpreted as Judgemental

It was agreed that all women feel emotionally fragile during the postpartum period and therefore, receiving encouragement is important. PPI collaborators spoke about the value of a weight management intervention that adopts a friendly and personal approach and shows understanding of the barriers a mother must overcome to eat better and be more physically active. Similarly, they felt that any text that could be perceived as patronising or passing judgement would hinder participants' motivation and engagement with the intervention and ultimately, their progress. These factors had been carefully considered during the development of the initial library of text messages used in the pilot study, however it was important to gain further feedback from PPI representatives on any instances where the language was not as sensitive as it should be. Women also advised that capital letters can often strike a demanding tone that should be avoided e.g. ‘WATCH what you eat’.

### 3.6 Avoid Downplaying the Effort Required for Weight Loss

PPI collaborators pointed out that a few text messages risked being perceived as oversimplifying the weight management process. They felt that downplaying the difficulty of weight loss may discourage any study participant who cannot achieve what the text message describes and may make them feel like they are failing. This had been carefully considered during the development of the initial library of text messages used in the pilot study, but it was important to refine any messages that could be interpreted as discouraging.

### 3.7 Avoid Sensitive Terms

A few PPI collaborators highlighted words or phrases which could have a negative connotation for a mother who is trying to manage their weight, such as ‘thin’ or ‘bingo wings’ (when recommending a targeted workout). They identified such terms that may make the text recipients feel uncomfortable and suggested they were removed and replaced. Additionally, women disliked the phrase ‘*Suffering from Busy Mum Syndrome?*’, as they felt it implied that being a mother was associated with being unwell. Upon receiving this feedback, the research team changed the phrase to ‘*Every mum is a busy mum!*’.

### 3.8 Include More Humour and Emojis

PPI collaborators sought humour and regarded it as an important element of the text messages. Although the messages did contain emojis, women wanted to see more used as they felt that this visual element helps to break up the text and further highlights the humour included in the messages. As it was pointed out, ‘*sometimes an emoji is a complete reply*’.

Even though humorous messages were generally positively received, it became obvious that humour is culturally specific and not everyone appreciated the same jokes. The sessions with PPI representatives from London and Bradford revealed disagreements in what mothers from each area considered to be funny. At the same time, participants acknowledged that everyone had a different sense of humour and, despite not finding some messages funny, they could appreciate that other women might. In such cases, the research team decided to maintain the humour as long as the messages were generally acceptable and understood by women.

### 3.9 Consider More Culturally Appropriate Suggestions for Food and Exercise Tips

PPI collaborators advised on the relevance of snack ideas, recipes, food preparation and work‐out ideas, and suggested alternatives when these did not resonate with or conflicted with their cultural backgrounds. They also gave an insight into social occasions or religious holidays that were related to food in their own cultures. Specifically, in terms of being active after birth, South Asian participants from Bradford gave useful insights into the expectations of their community on how active a mother should be in the early postpartum life: *‘… coming from a Pakistani background, the type of messages that you have, for example, “get up and go out for a walk,” that would not be received very well and it would be seen as far too much for a mom…*’.

### 3.10 If a Message Urges Participants to Act, Clearly State Its Purpose

Some texts invited participants to self‐reflect and respond to a question e.g. what motivated you to take part in the SMS study? Some texts prepared them for what would follow in subsequent text messages. PPI collaborators highlighted that such texts needed to include clear instructions on the purpose of the message, what participants were being asked to do or what they should expect from future texts.

## 4 Discussion

This PPI work aimed to review and adapt the content of a text message library to support diet and physical activity behaviour change for weight loss and weight loss maintenance, so that it is relevant and appropriate for a geographically, socioeconomically and culturally diverse population of women in the postpartum period. The text message library is the core component of the SMS intervention, which will be delivered to all participants of the SMS full trial. It was previously developed with PPI and showed high feasibility and acceptability in a pilot RCT in one part of the UK [20]. This cultural and regional adaptation was conducted in preparation for conducting a UK‐wide RCT to evaluate the effectiveness and cost‐effectiveness of the SMS intervention. In this PPI collaboration we worked alongside women in the postpartum period using approaches that considered their life circumstances and engaging in a way that suited their busy schedules. The PPI discussions focused on the length of the text messages, their language and clarity, their tone, as well as on the cultural relevance of the dietary and physical activity advice provided. None of the text messages needed major re‐writing.

In Bradford and Scotland, working with established PPI networks and research advisory forums facilitated direct access to individuals experienced in collaborating with researchers. In London, in the absence of such networks, community groups and networks that had a focus on motherhood were targeted for PPI recruitment. Engaging with existing networks was more time efficient and resulted in recruitment of more PPI collaborators (eight PPI representatives in Bradford and seven in Scotland compared with four in London) aligning with similar experiences reported in the literature [32]. This demonstrates the importance of building and sustaining trusted networks of members of the public who are interested in contributing to research, the value of which has been previously described by researchers who have successfully established community advisory groups for health research [33, 34]. However, sustaining networks in the longer‐term can be challenging for postpartum research as it focuses on a very specific time in a woman's life.

For this cultural adaptation, our PPI representatives came from diverse ethnic and socioeconomic groups from England and Scotland. They expressed a desire for an initiative like SMS and commented positively on the supportive and encouraging tone of the study text messages, as well as the way the messages recognised their busy lives and the challenges associated with this when it comes to self‐care. Their feedback reinforced the benefit of the initial PPI work [20] where extensive care was taken to ensure messages were framed appropriately using sensitive language, which is especially important at this physically, psychologically and socially challenging and demanding time in a woman's life.

Suggestions for adapting the text messages were minor and related to reducing the length of longer messages, avoiding judgemental and stigmatising language, including more humour and refraining from the use of colloquial phrases which may not be understood. These factors had been central considerations in the initial development of the text message library [20], but this PPI work highlighted areas that needed further attention and refining to ensure wider acceptability and relevance. According to Resnicow and colleagues, language is an essential characteristic of a target population that should be taken into account when adapting the information of a public health intervention [35]. It was evident during the initial PPI work [20] and the work described here that every word is important when writing short messages and changing just a word or two can completely change the meaning of a message and how it is received by the target group. In addition to this, researchers are recommended to gain a better understanding of deeper structures that influence the health behaviours of different ethnic and sociodemographic subpopulations (e.g. norms and beliefs), to make health promotion research culturally sensitive and appropriate [35]. During this study we sought feedback on messages that covered areas that are highly relevant to tradition and cultural identity, such as cooking preparations and social gatherings. Discussions during the PPI sessions highlighted the role of the extended family in different cultures and how these influenced their way of eating. This was particularly evident among the South Asian community, in accordance with previous qualitative explorations highlighting that South Asian women living in the UK have very distinct ideas regarding food in social situations and the types of food they are expected to offer as hostesses compared to women of White ethnicity [36]. It is important that these cultural nuances are understood and incorporated into interventions.

A weight management intervention that is relevant to all women across the UK should include dietary and activity suggestions that are appropriate for a wide range of ethnic and cultural backgrounds and for all budgets. PPI collaborators made no comments or objections around the affordability of following the dietary and exercise advice in the SMS text messages. As previously mentioned, the initial text message library had been co‐produced with mothers, who had already advised on including financially viable snack and meal options, as well as opportunities for physical activities with little or no cost. Additionally, the researchers involved in developing and adapting the library of text messages have experience in public health with a focus on food security and the challenges faced by economically disadvantaged families. Overall, reflecting on previous PPI work and research experience means that themes such as food affordability and easy access to nutritious meals when time and other resources are limited were prioritised within the SMS text messages.

## 5 Strengths and Weaknesses

The PPI group sessions took place online, enabling broad geographical representation and providing flexibility for mothers to join from home. Safety was also a consideration, as this study commenced a few months after the final COVID‐19 lockdown restrictions were lifted (May 2021) at a time when many people were still cautious about face‐to‐face meetings. However, meeting virtually may have diminished some aspects of in‐person interaction, such as observing body language.

During recruitment for this PPI work, we invited mothers who at the time had a child below the age of 2 years and had struggled with their weight. PPI collaborators were not formally screened for specific eligibility criteria (e.g., weight, height, or child's exact age), as participants of the SMS full trial would be, i.e. living with a BMI ≥ 25 kg/m^2^ and having had a child within the last 2 years. However, all collaborators shared lived experiences of postpartum weight challenges.

Participants included both experienced PPI representatives and those new to research involvement. To ensure inclusivity and similar levels of engagement, great care was taken to ensure everyone received clear communication about the scope of the PPI activities, felt comfortable with the PPI process and was encouraged to provide their thoughts and opinions. Ultimately, we observed no notable differences in the level of engagement, feedback or participation during the PPI sessions from more experienced versus newer PPI collaborators.

Overall, this PPI collaboration to culturally adapt the text message library extended the UK‐wide SMS RCT timeline by 6 months and came with associated financial costs. However, this engagement and investment has improved the wider relevance of the SMS text message library, reducing the risk of resource wastage during the UK‐wide RCT [27]. This underscores the value of allocating resources to meaningful PPI, a priority that is acknowledged and fully supported by the study's funder, the National Institute for Health Research.

## 6 Conclusion

The cultural adaptation of the content of the SMS intervention was conducted using methods that worked for a postpartum group of mothers and had been used in the initial development of the text message library. PPI provided helpful suggestions for the cultural and regional adaptation of the SMS text messages. These suggestions increased the clarity of the language used in the texts, with removing and rephrasing some of the colloquialisms that are not widely understood and used across the UK. PPI collaborators also fostered the encouraging and humorous tone of the texts, whilst providing insightful suggestions to make the advice on exercise, meals and snacks and food‐related social occasions relevant to women across the UK socio‐cultural spectrum. Overall, minor modifications were required to the messages, but this study demonstrated that even small changes to wording can contribute to how written information is received by the target audience. It is hoped that this study will help to ensure that the messages are appropriate and relevant to mothers from a wide range of backgrounds. This will be formally tested in an ongoing UK‐wide RCT [21].

## Author Contributions

Conceptualization: Michelle C. McKinley, Sally Bridges, and Eleni Spyreli. Methodology: Eleni Spyreli, Lizzie Caperon, Emma Ansell, Sara Ahern, Sally Bridges, and Michelle C. McKinley. Formal analysis: Eleni Spyreli and Michelle C. McKinley. Investigation: Eleni Spyreli, Lizzie Caperon, Emma Ansell, Sara Ahern, and Sally Bridges. Resources: Michelle C. McKinley and Sally Bridges. Data curation: Eleni Spyreli and Lizzie Caperon. Writing – original draft preparation: Eleni Spyreli and Michelle C. McKinley. Writing – review and editing: Eleni Spyreli, Lizzie Caperon, Emma Ansell, Sara Ahern, Sally Bridges, Elinor Coulman, Stephan U. Dombrowski, Frank Kee, Jayne V. Woodside, Dunla Gallagher, and Michelle C. McKinley. Supervision: Michelle C. McKinley, Sally Bridges. Project administration: Michelle C. McKinley and Sally Bridges. Funding acquisition: Michelle C. McKinley, Sally Bridges, Elinor Coulman, Stephan U. Dombrowski, Frank Kee, Jayne V. Woodside, and Dunla Gallagher. All authors have read and agreed to the published version of the manuscript.

## Ethics Statement

Approval from a research ethics committee was not required for Personal and Public Involvement work for the cultural adaptation of the content of the intervention. No research data was collected.

## Conflicts of Interest

J.V.W. is a trustee of the Nutrition Society for which she does not receive payment.

## Supporting information


**Figure 1:** Posters used for recruitment to PPI activities.

**Figure 2:** Participant Information Sheet sent to women who expressed interest in participating in the PPI activities.

**Figure 3:** Examples of text messages reviewed at the first stage of the PPI work.

**Figure 4:** Example of feedback from a PPI participant who reviewed the entire library of the Supporting MumS text messages.

**Supplementary Table 1:** Guidance for reporting involvement of PPI representatives (GRIPP2 checklist)^1^.

## Data Availability

The data that support the findings of this study are available on request from the corresponding author. The data are not publicly available due to privacy or ethical restrictions.
