# Supplementary material for: Adaptation of the Content of a Behavioural Text Message Delivered Weight Management Intervention for a Socio‐Culturally and Geographically Diverse Population of Postpartum Women in the UK: The Supporting MumS (SMS) Intervention
Source: Health Expect. 2025 Aug 6;28(4):e70368. doi: 10.1111/hex.70368 (PMC12326423; doi:10.1111/hex.70368)
Supplement: Supplementary file 1 — Figure 1: Posters used for recruitment to PPI activities. [file HEX-28-e70368-s004.docx]

Supplementary Table 1. Guidance for reporting involvement of PPI representatives (GRIPP2 checklist) ^1^

| Section and topic | Item |
| --- | --- |
| 1: Aim | Our aim was to review and adapt the content of a library of text messages to support diet and physical activity behaviour change for weight loss and weight loss maintenance for a socio-culturally and geographically diverse population of postpartum women. |
| 2: Methods | Consistent with the population who would be recruited for the subsequent effectiveness trial, PPI recruitment focused on mothers who had had a child in the last two years and struggled with their body weight. PPI representatives were identified through existing PPI networks and community groups. PPI activities were conducted in online group sessions and on a one-to-one basis through email correspondence. Power point presentations and a word document specifically designed to gather representatives’ feedback were used for the group sessions and individual collaboration respectively. Sound recordings and representatives’ notes were compiled as the output of the PPI discussions. The recordings were destroyed when complete notes were compiled for each session. Τhe notes were then reviewed to identify common areas for amendment that were then applied to the library of text messages. |
| 3: Study results | PPI representatives confirmed the need for programmes like Supporting MumS and commented favourably on the tone and content of the text messages. Minor changes were suggested to improve the length and clarity of the messages, and to ensure that their tone was encouraging and humorous when appropriate. Colloquialisms and sensitive terms were identified within the texts and were replaced by alternative wordings. Dietary and exercise alternatives were suggested when the original ones were not culturally relevant for all UK women. |
| 4: Discussion and conclusions | The PPI work offered valuable insights into ways of enhancing the socio-cultural and regional relevance of a library of text messages to support diet and physical activity behaviour change for weight loss and weight loss maintenance in the postpartum period. |
| 5: Reflections/critical perspective | Our recruitment process employed different approaches with variable levels of success, highlighting the importance of building and maintaining strong networks with the community prior to conducting PPI. Additionally, maintaining flexibility when engaging and working with the PPI collaborators is key. It is important to choose appropriate methods that take into consideration the life circumstances of PPI collaborators and maximise opportunities for engagement. PPI should be an integral part of all research, but it is a time- and resource-intensive process that should be accounted for when planning research and recognised and resourced by research funders. |

Abbreviations: RCT – randomised controlled trial, PPI – personal and public involvement

^1^ *Staniszewska S, Brett J, Simera I, Seers K, Mockford C, Goodlad S, et al. GRIPP2 reporting checklists: tools to improve reporting of patient and public involvement in research. bmj. 2017 Aug 2;358.*
