# Supplementary figures and images for: Adaptation of the Content of a Behavioural Text Message Delivered Weight Management Intervention for a Socio‐Culturally and Geographically Diverse Population of Postpartum Women in the UK: The Supporting MumS (SMS) Intervention
Source: Health Expect. 2025 Aug 6;28(4):e70368. doi: 10.1111/hex.70368 (PMC12326423; doi:10.1111/hex.70368)

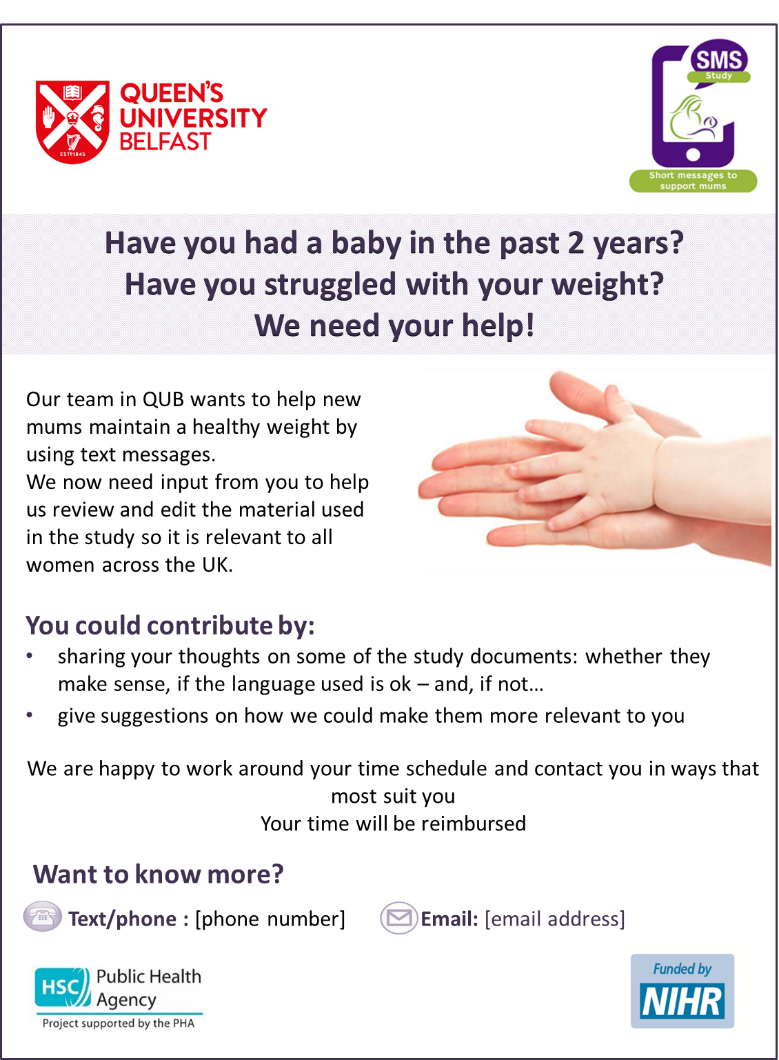
Figure 1. Posters used for recruitment to PPI activities


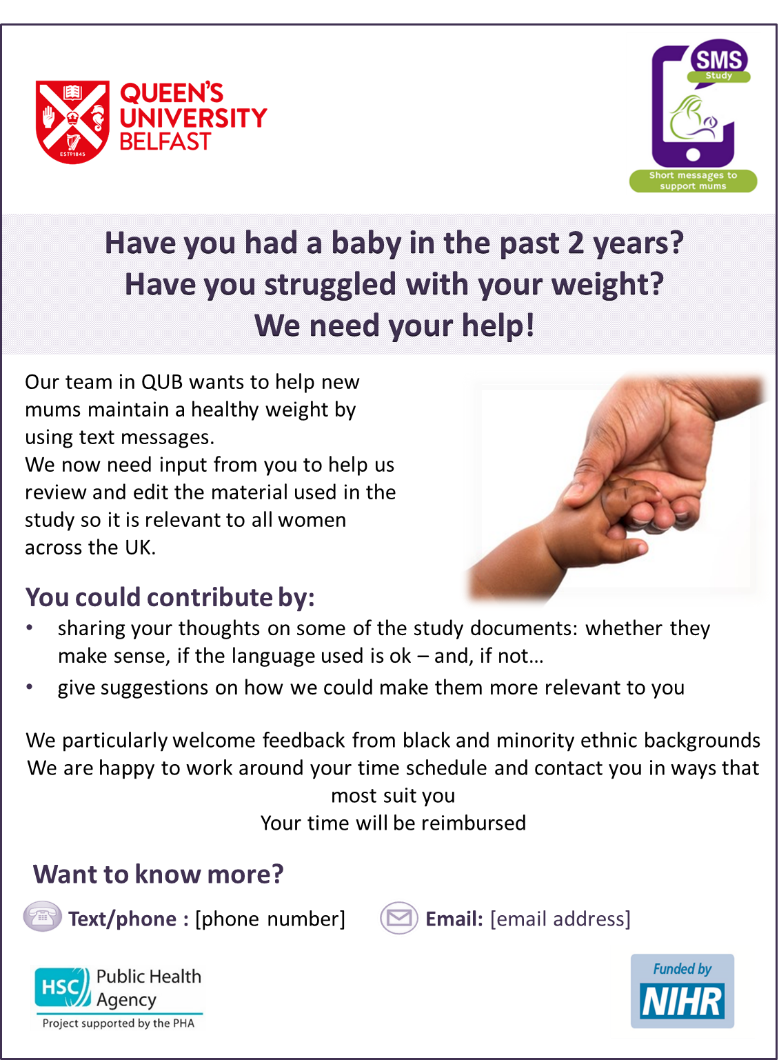


s

Supplement: Supplementary file 2 — Figure 2: Participant Information Sheet sent to women who expressed interest in participating in the PPI activities. [file HEX-28-e70368-s005.docx]

Figure 3. Examples of text messages reviewed at the first stage of the PPI work


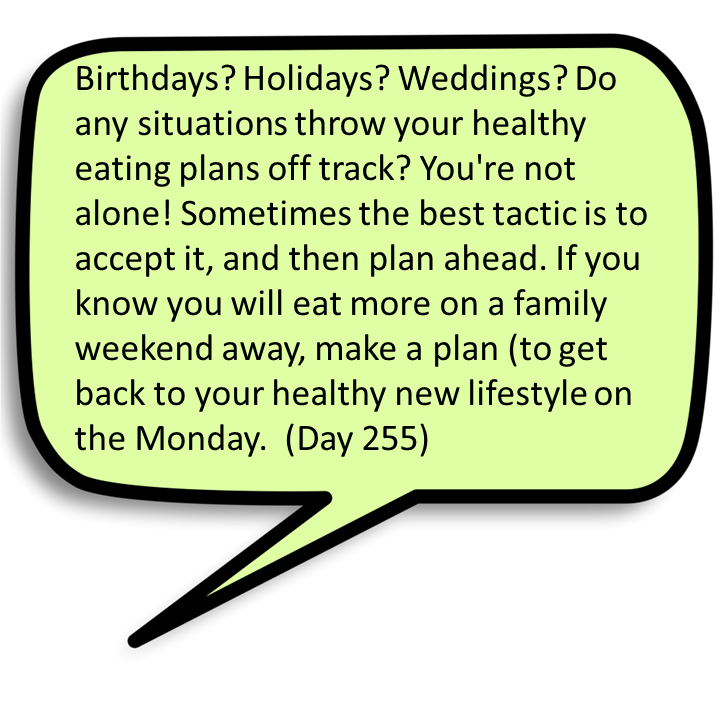


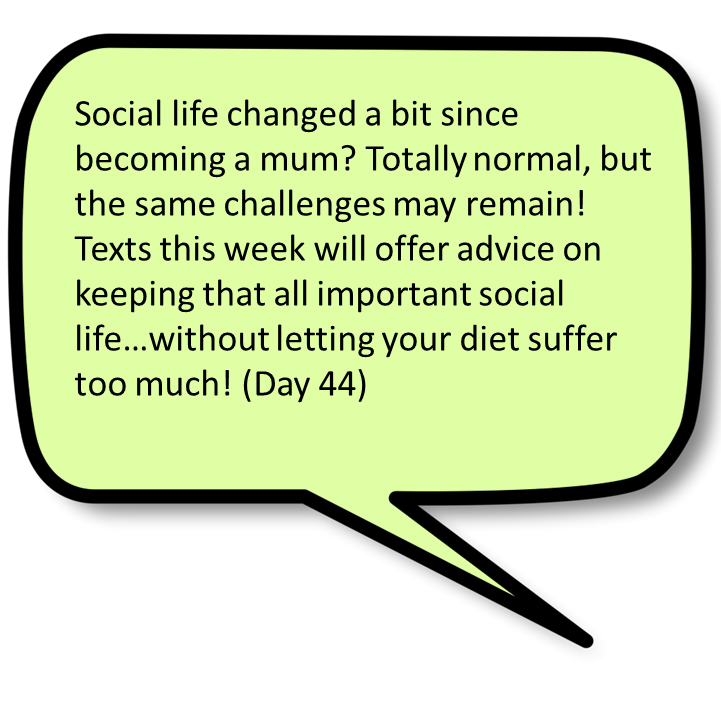

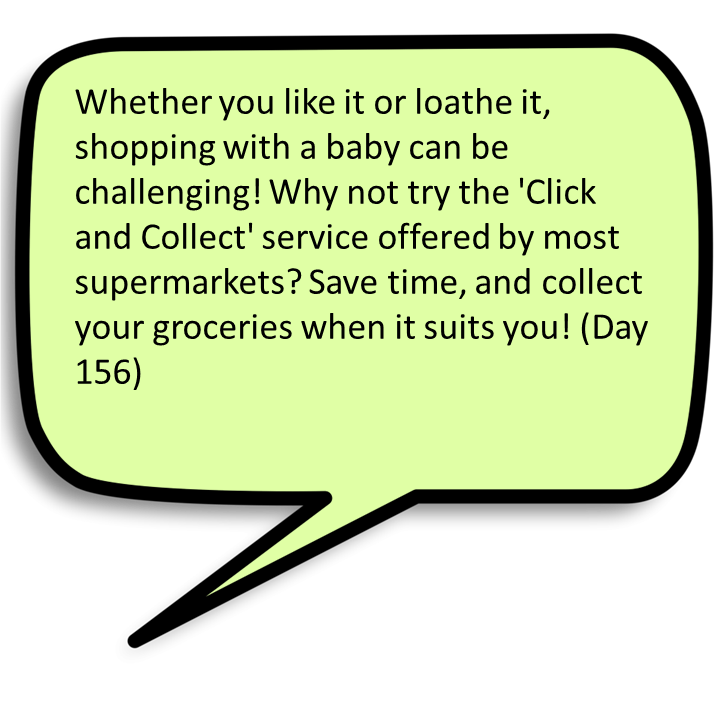

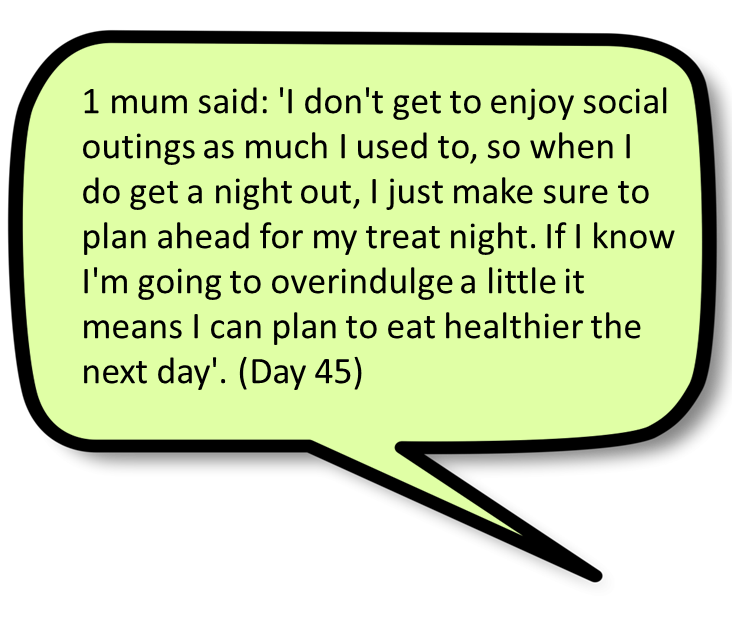

Supplement: Supplementary file 4 — Figure 4: Example of feedback from a PPI participant who reviewed the entire library of the Supporting MumS text messages. [file HEX-28-e70368-s003.docx]
