# Supplementary material for: Adaptation of the Content of a Behavioural Text Message Delivered Weight Management Intervention for a Socio‐Culturally and Geographically Diverse Population of Postpartum Women in the UK: The Supporting MumS (SMS) Intervention
Source: Health Expect. 2025 Aug 6;28(4):e70368. doi: 10.1111/hex.70368 (PMC12326423; doi:10.1111/hex.70368)
Supplement: Supplementary file 3 — Figure 3: Examples of text messages reviewed at the first stage of the PPI work. [file HEX-28-e70368-s002.docx]

Figure 2. Participant Information Sheet sent to women who expressed interest in participating in the PPI activities.


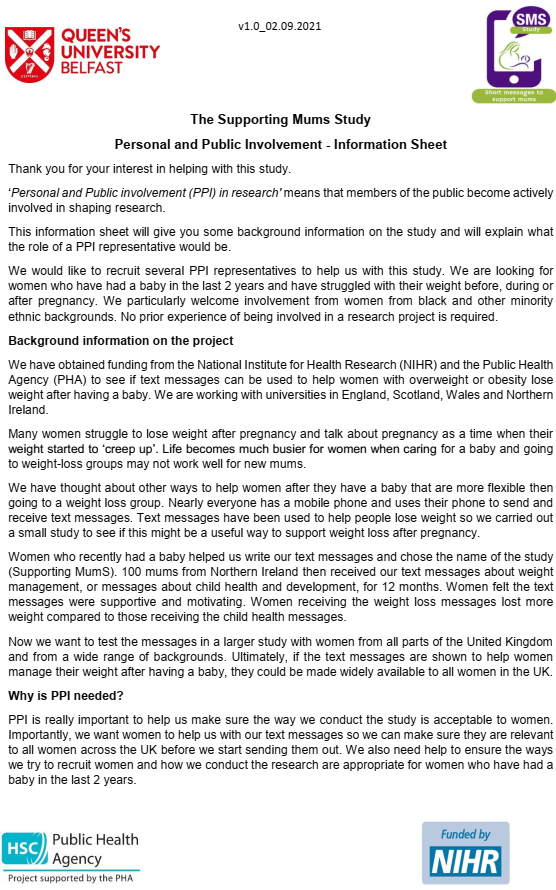


Figure 2. Participant Information Sheet sent to women who expressed interest in participating in the PPI activities. (Continued)


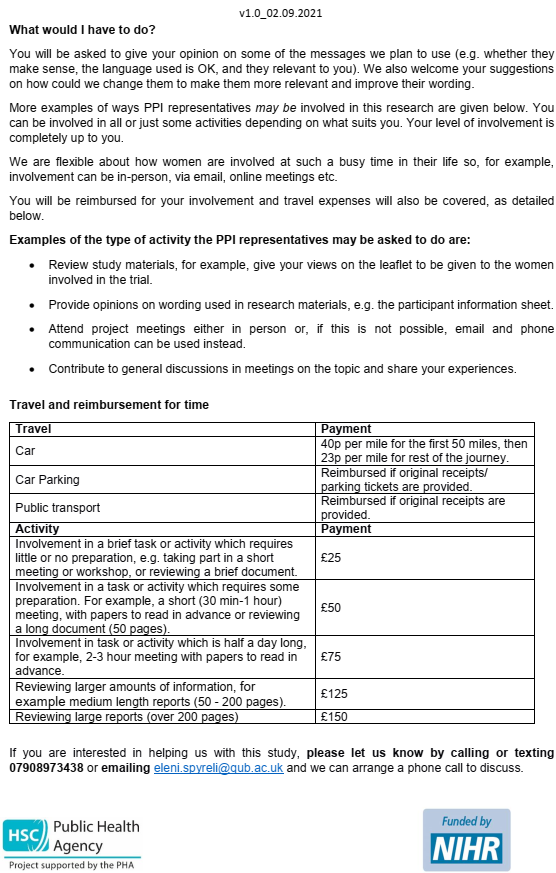


[researcher’s phone number and email address]
