# Supplementary material for: Adaptation of the Content of a Behavioural Text Message Delivered Weight Management Intervention for a Socio‐Culturally and Geographically Diverse Population of Postpartum Women in the UK: The Supporting MumS (SMS) Intervention
Source: Health Expect. 2025 Aug 6;28(4):e70368. doi: 10.1111/hex.70368 (PMC12326423; doi:10.1111/hex.70368)
Supplement: Supplementary file 5 — Supplementary Table 1: Guidance for reporting involvement of PPI representatives (GRIPP2 checklist)1. [file HEX-28-e70368-s001.docx]

Figure 4. Example of feedback from a PPI participant who reviewed the entire library of the Supporting MumS text messages
